# An Optimized Pedestrian Inertial Navigation Method Based on the Birkhoff Pseudospectral Method

**DOI:** 10.3390/s26061850

**Published:** 2026-03-15

**Authors:** Zihong Zhang, Dangjun Zhao, Di Tian

**Affiliations:** School of Automation, Central South University, Changsha 410083, China; zzh819@csu.edu.cn (Z.Z.); tiandi_csu@csu.edu.cn (D.T.)

**Keywords:** pedestrian navigation, Birkhoff pseudospectral method, strapdown inertial navigation

## Abstract

Pedestrian inertial navigation is a pivotal technology for achieving seamless indoor and outdoor positioning. Traditional methods based on the Extended Kalman Filter (EKF) suffer from cumulative errors induced by inertial measurement unit (IMU) noise, which severely degrade the accuracy of pedestrian trajectory estimation over long durations. To address this critical limitation, a post-processing trajectory optimization approach for pedestrian inertial navigation based on the Birkhoff pseudospectral method is proposed in this paper. Leveraging the initial attitude and position estimates derived from the Zero-Velocity Update (ZUPT) technique and the EKF framework, the proposed method first parameterizes continuous-time acceleration measurements by adopting Chebyshev nodes as collocation points, and then formulates and solves the trajectory optimization problem via a Birkhoff pseudospectral framework, which effectively suppresses noise interference from the IMU accelerometer. Simulation experiments validate the superior noise suppression capability of the proposed algorithm. Furthermore, physical experiments conducted with a foot-mounted IMU demonstrate that the final position error is reduced by approximately 90% in comparison with the traditional EKF-based method.

## 1. Introduction

With the advancement of technology, pedestrian navigation systems have been widely deployed in various application domains, including indoor positioning, emergency rescue, and autonomous mobile robotics [1]. Pedestrian navigation systems can be categorized into non-autonomous and autonomous types according to their dependencies on external reference signals. Non-autonomous navigation relies on external reference signals from the Global Navigation Satellite System (GNSS) or other pre-deployed infrastructure (e.g., WiFi and Bluetooth) [2]. In contrast, autonomous navigation systems operate purely on onboard sensors, and inertial measurement units (IMUs) serve as their core sensing component. Among autonomous navigation systems, IMU-based pedestrian navigation systems (PNSs) play an irreplaceable role in GNSS-denied environments (e.g., indoor spaces, underground tunnels, and dense urban canyons) [3].

Autonomous IMU-based PNSs can be further divided into foot-mounted and non-foot-mounted configurations based on on-body sensor placement [4]. Non-foot-mounted systems typically adopt a step-and-heading system (SHS) approach, which detects gait cycles, estimates step length and heading, and then calculates the positional changes of pedestrians accordingly. Although this configuration allows the IMU to be worn on various body locations (e.g., the waist, wrist, or chest), it relies on idealized walking models and fails to fully exploit the rich acceleration and angular velocity information provided by the IMU [5]. In recent years, deep learning-based methods have been proposed for step length and heading angle estimation, which improve the adaptability of SHS to different gait patterns and motion states [6].

In contrast, foot-mounted pedestrian navigation systems track the real-time position and orientation of pedestrians by integrating measurements from gyroscopes and accelerometers. A key premise of this integration is that the foot-mounted IMU is generally treated as a rigid body [7] since the sensor is securely attached to the foot without relative sliding or displacement during gait, and the foot itself exhibits high mechanical stability with minimal deformation in typical walking scenarios. This fundamental assumption bridges foot-mounted PNS to Strapdown Inertial Navigation System (SINS) theory [8]. With the continuous development of Micro-Electro-Mechanical System (MEMS) technology, low-cost, miniaturized IMUs have been widely integrated into consumer electronics such as smartphones and wearable devices, providing a solid hardware foundation for the practical application of foot-mounted pedestrian navigation technologies [9].

However, achieving high-accuracy pedestrian positioning with MEMS IMUs remains a formidable challenge because the sensors are inherently affected by random noise, bias drift, and scale-factor errors [10]. In PNSs, the high-precision estimation of velocity and position is a core determinant of overall system performance. Traditional positioning methods, which conform to the core logic of SINSs, typically obtain velocity and position sequentially by double integrating the specific force measured by accelerometers [11]. Nevertheless, these approaches suffer from two inherent defects: (1) the noise of MEMS IMUs is accumulated and amplified during the integration process, leading to position errors that diverge at a rate proportional to the cube of time; and (2) in highly dynamic motion scenarios, residual errors in gravity compensation further exacerbate the inaccuracies of velocity and position estimation [3,12]. The existing research indicates that for low-cost MEMS IMUs, the positioning errors can reach 5% to 10% of the total traveled distance even over short periods, and such error divergence becomes more pronounced during complex motions such as running or variable-speed walking [13].

To enhance the accuracy of traditional integration methods, researchers have turned to approaches based on kinematic modeling and parametric fitting. A typical category of methods uses polynomials or other basis functions to locally fit specific force and angular velocity signals within sampling intervals. In [14], the authors constructed a continuous-time motion model, thereby replacing the approximate assumptions (e.g., piecewise constant or linear approximation) inherent in traditional methods. Yang et al. [15] proposed a velocity estimation algorithm based on specific-force polynomial modeling. By optimizing polynomial coefficients and constructing a high-precision attitude matrix, their method achieved significant improvement in velocity estimation accuracy within the geographic coordinate frame. On the other hand, Zhu [16] and Wu et al. [17] employed Chebyshev polynomials to transform the continuous-time navigation-state estimation problem into an optimization problem for polynomial coefficients, realizing error minimization through coefficient optimization. Such methods can effectively suppress dynamic error terms such as sculling errors, offering a promising solution for inertial navigation in highly dynamic environments.

In fact, the methods in [16,17] adopted a collocation strategy similar to the pseudospectral method widely used in optimal control and trajectory optimization. The core idea of the traditional pseudospectral method is to parameterize the state trajectory using global interpolating polynomials at a series of collocation points, which transforms continuous differential equation constraints into algebraic constraints and ultimately converts the original problem into a nonlinear programming (NLP) problem [18,19,20]. Unlike the traditional pseudospectral method, the Birkhoff pseudospectral method proposed in [21] leverages the characteristic of Birkhoff polynomials that perform interpolation based on the original function values at the boundaries and the derivatives at the internal collocation points. This enables it to conveniently incorporate the position constraints at the trajectory endpoints into the optimization problem, reduce the condition number of the transformed problem, and facilitate rapid solution [22].

Inspired by the collocation strategy of [17] and the Birkhoff pseudospectral method in [21], this paper introduces the Birkhoff pseudospectral method for solving the trajectory estimation problem of pedestrian inertial navigation, focusing on how to utilize its global approximation framework for high-precision estimation and optimization of pedestrian motion trajectories. To the best of our knowledge, there are no reported papers that apply the Birkhoff pseudospectral method to the solution of pedestrian navigation trajectory optimization. The main contributions of this paper lie in the following two aspects: (1) a two-step algorithm framework is constructed, which takes the bias-corrected state sequence from Zero Velocity Update (ZUPT)-aided Extended Kalman Filter (EKF) as initial input and realizes global trajectory optimization through window division based on gait cycles; (2) the Birkhoff pseudospectral method is used to model the pedestrian trajectory optimization problem with a carefully designed collocation strategy and objective function to ensure optimization accuracy and robustness.

The remainder of this paper is organized as follows. Section 2 outlines the theoretical foundations, including the principles of SINS, ZUPT, and EKF. Section 3 details the proposed trajectory optimization framework based on the Birkhoff pseudospectral method. Section 4 and Section 5 present the simulation and physical experiments conducted to validate the performance of the proposed algorithm, respectively; finally, Section 6 concludes the paper and discusses future research directions.

## 2. Theoretical Basis

### 2.1. Principles of SINS

The core principle of SINS is to estimate the navigation states of a carrier in real time by solving kinematic equations based on the specific-force and angular velocity information output by a six-axis IMU, which integrates three orthogonal accelerometers for specific-force detection and three orthogonal gyroscopes for angular velocity measurement. For the clarity of subsequent derivation, three coordinate frames are defined in this paper: (1) the body frame (denoted as b-frame), which is rigidly fixed to the IMU and moves with the carrier; (2) the navigation frame (denoted as n-frame), which adopts the local-level frame (east–north–up, ENU) for pedestrian navigation; (3) the inertial frame (denoted as i-frame), which takes the Earth-centered inertial (ECI) frame as the reference [23].

For navigation solutions over short time intervals, the Earth’s rotation and the curvature of the Earth can be neglected, and the system dynamics can be described by the following set of differential equations:

(1) $${\dot{q}}_{b}^{n}=\frac{1}{2}q_{b}^{n}⊗\omega_{ib}^{b}$$

(2) $${\dot{v}}^{n}=C_{b}^{n}f_{ib}^{b}+g^{n}$$

(3) $${\dot{p}}^{n}=v^{n}$$

where $\omega_{ib}^{b}$ denotes the true angular velocity of the b-frame with respect to the i-frame. $C_{b}^{n}$ denotes the rotation matrix from the b-frame to the n-frame. $f_{ib}^{b}$ denotes the true specific force acting on the carrier in the b-frame. $v^{n}$ denotes the velocity vector of the carrier in the n-frame. $g^{n}$ and $p^{n}$ denote the gravity acceleration vector in the n-frame and the position vector of the carrier in n-frame, respectively. $q_{b}^{n}$ represents the attitude quaternion from the b-frame to the n-frame. The operator ⊗ denotes quaternion multiplication, which for two quaternions P and Q is defined as

(4) $$P⊗Q=M_{P}\left[\begin{array}{c} q_{0}\\ q_{v} \end{array}\right]=M_{Q}^{\prime }\left[\begin{array}{c} p_{0}\\ p_{v} \end{array}\right]$$

Two quaternion multiplication matrices $M_{P}$ and $M_{Q}^{\prime }$ are defined respectively as

(5) $$M_{P}=\left[\begin{array}{cc} p_{0} & -p_{v}^{T}\\ p_{v} & p_{0}I+(p_{v}\times ) \end{array}\right],M_{Q}^{\prime }=\left[\begin{array}{cc} q_{0} & -q_{v}^{T}\\ q_{v} & q_{0}I-(q_{v}\times ) \end{array}\right]$$

where I represents a 3×3 identity matrix. The skew-symmetric matrix (⋅×) is defined to satisfy the cross-product relation x×y=(x×)y. The transformation relationship between the attitude matrix $C_{b}^{n}$ and the attitude quaternion is given by

(6) $$C_{b}^{n}=I+2q_{0}(q_{v}\times )+2{\left(q_{v}\times \right)}^{2}$$

### 2.2. Principles of the EKF

The EKF provides a widely adopted framework for real-time state estimation in nonlinear systems. In this work, an error-state EKF is employed to correct the drifting inertial navigation solution by fusing IMU measurements with external observations. Its error-state vector generally includes error terms such as attitude, velocity, position, and sensor biases, which are specifically defined as [24]

(7) $$\delta x={\left[\begin{array}{cc} \delta p & \begin{array}{cccc} \delta v & \delta \theta & \delta b_{a} & \delta b_{\omega } \end{array} \end{array}\right]}^{T}\in {\mathbb{R}}^{15}$$

where δp denotes the position error vector in the navigation frame, δv is the velocity error vector, δθ represents the attitude error vector, and $\delta b_{a}$ and $\delta b_{\omega }$ are the measurement bias error vectors of the accelerometer and gyroscope, respectively.

The continuous-time error dynamics are linearized as:

(8) $$\delta \dot{x}=F\delta x+Gw,w∼N(0,Q_{c})$$

where F is the continuous-time state-transition matrix, G is the noise input matrix, and $Q_{c}$ is its corresponding power spectral density matrix.

Discretizing over a sampling interval Δt yields:

(9) $$\delta x_{\left.k+1\right|k}=\phi_{k}\delta x_{\left.k\right|k}+w_{k},w_{k}∼N(0,Q_{k})$$

where $\phi_{k}=\exp(F\Delta t)$ is the state-transition matrix and $Q_{k}$ is the discrete-time process noise covariance. The error covariance is propagated as:

(10) $$P_{\left.k+1\right|k}=\phi_{k}P_{\left.k\right|k}{\phi_{k}}^{T}+Q_{k}$$

where P is the estimation error covariance matrix. When external measurements are available, the filter corrects the error state using a linearized observation model:

(11) $$z_{k}=H\delta x_{k}+n_{k},n_{k}∼N(0,R_{k})$$

where $z_{k}$ is the measurement residual vector, and H is the measurement Jacobian. $R_{k}$ is the measurement noise covariance matrix. The Kalman gain $K_{k}$ is computed in the usual way, and the error-state estimate and its covariance are updated.

The EKF operates recursively. During the prediction phase, the IMU measurements propagate the nominal state via the nonlinear kinematic equations, while the error-state covariance is updated linearly using the system Jacobian. In the update phase, the zero-velocity observations identified by ZUPT serve as measurement constraints. The Kalman gain is computed to fuse this information, correcting the error-state estimates and calibrating the sensor biases in real-time.

Thus, the EKF leverages the periodic zero-velocity constraints to suppress the cubic drift of the pure inertial solution and provides a smoothed, bias-corrected state sequence [25]. This sequence serves as the initial trajectory and prior information for the subsequent window-based Birkhoff pseudospectral optimization.

### 2.3. ZUPT Algorithm

ZUPT is a core method for suppressing error accumulation in inertial navigation systems, and its effectiveness hinges on the accurate detection of zero-velocity intervals. The principle of ZUPT is rooted in the physical constraint that, during the stance phase of pedestrian gait, the foot-mounted IMU is momentarily stationary relative to the ground, yielding a true velocity of zero. By detecting these intervals, ZUPT injects a “virtual measurement” of zero velocity into the navigation filter, which corrects accumulated velocity, position, and attitude errors propagated from inertial sensor noise and bias.

The Generalized Likelihood Ratio Test (GLRT) provides a statistically rigorous and practically implementable framework for this detection task [26]. It formulates zero-velocity detection as a hypothesis-testing problem. Under the null hypothesis $H_{0}$, the IMU measurements consist only of sensor noise:

(12) $$\left\{\begin{array}{c} y_{k}^{a}=g\frac{{\bar{y}}_{n}^{a}}{\left\|{\bar{y}}_{n}^{a}\right\|}+\varepsilon_{k}^{a},\varepsilon_{k}^{a}∼N\left(0,\sigma_{a}^{2}I_{3}\right)\\ y_{k}^{\omega }=\varepsilon_{k}^{\omega },\varepsilon_{k}^{\omega }∼N\left(0,\sigma_{\omega }^{2}I_{3}\right) \end{array}\right.$$

where $y_{k}^{a}$ and $y_{k}^{\omega }$ represent the three-axis acceleration and three-axis angular velocity output by the IMU, respectively; ${\bar{y}}_{n}^{a}$ is the mean vector of accelerometer measurements within the corresponding detection window. $\sigma_{a}^{2}$ and $\sigma_{\omega }^{2}$ are the variances of accelerometer measurement noise and gyroscope measurement noise, respectively, which are calibrated using Allan variance analysis. Under the alternative hypothesis $H_{1}$ (foot in motion), the measurements contain both the true motion signals and sensor noise.

Assuming independent and identically distributed samples, the log-likelihood ratio for a window of length W is:

(13) $$\log\Lambda =\frac{1}{W}\sum_{k=n}^{n+W-1}\left(\log\;p(\left.y_{k}^{a},y_{k}^{\omega }\right|H_{0})-\log\;p(\left.y_{k}^{a},y_{k}^{\omega }\right|H_{1}\right)$$

Substituting the Gaussian densities and simplifying under the assumption that $H_{1}$ has no deterministic structure (i.e., the signal can take any value), the GLRT statistic reduces to:

(14) $$T(z_{n}^{a},z_{n}^{\omega })=\frac{1}{W}\sum_{k=n}^{n+W-1}\left(\frac{1}{\sigma_{a}^{2}}{\left\|y_{k}^{a}-g\frac{{\bar{y}}_{n}^{a}}{\left\|{\bar{y}}_{n}^{a}\right\|}\right\|}^{2}+\frac{1}{\sigma_{\omega }^{2}}{\left\|y_{k}^{\omega }\right\|}^{2}\right)$$

The test compares T to a threshold ς, declaring a zero-velocity interval if T≤ς.

In pedestrian navigation, foot-mounted or wearable IMU data exhibit distinct statistical characteristics between the stance and swing phases. During stance, accelerometer and gyroscope measurements can be approximated as zero-mean Gaussian noise. In contrast, swing-phase data includes deterministic signals from pedestrian dynamics alongside high-intensity random noise.

Compared to heuristic threshold or handcrafted feature-based methods, GLRT eliminates cumbersome parameter tuning. By maximizing the likelihood function, it automatically adapts to user-specific gait patterns, sensor installation variations, and environmental noise, thereby enhancing detection robustness, improving the detection probability, and reducing false-alarm rates. Integrating the GLRT into ZUPT robustly captures the brief static moments in the pedestrian stance phase [27,28], providing reliable zero-velocity observations for EKF measurement updates and effectively suppressing velocity and position error accumulation.

## 3. Pedestrian Navigation Algorithm Based on the Birkhoff Pseudospectral Method

The pseudospectral methods are a class of efficient numerical optimization methods, whose core lies in discretizing and controlling variables at a finite number of collocation points and using global orthogonal polynomials for interpolation, thereby transforming continuous-time optimal control problems into discrete NLP problems. The Birkhoff pseudospectral method is an important variant of the spectral method. It is inherently well-suited for handling various boundary conditions—a characteristic that makes it highly suitable for problems such as navigation trajectory optimization, which typically involve explicit initial values, terminal values, and process constraints [29].

**Figure 1:**
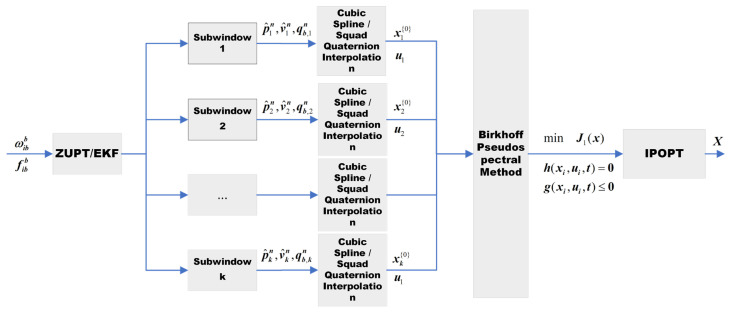
Flowchart of the proposed pedestrian navigation trajectory optimization algorithm based on the Birkhoff pseudospectral method.

Based on this, this paper designs a pedestrian navigation trajectory optimization method based on the Birkhoff pseudospectral method, whose overall workflow is shown in Figure 1.

### 3.1. Core Theory of the Birkhoff Pseudospectral Method

#### 3.1.1. Birkhoff Interpolation Principle

Birkhoff interpolation differs from Lagrange interpolation, which only constrains function values. It imposes mixed constraints on function values and multi-order derivatives, making it more suitable for complex dynamic systems. Let $\tau_{i}\in \pi^{N}(i=0,1,\ldots ,N)$ serve as collocation points, with the interpolation interval being $\left[\begin{array}{cc} -1 & 1 \end{array}\right]$. Given K+1 known real numbers $y_{i}^{\left(m\right)}$, determine the unique (highest) K-th order interpolation polynomial $f_{K}^{\left(m\right)}(\tau_{i})$ that exists and is unique, and satisfies the following relations:

(15) $$f_{K}^{\left(m\right)}(\tau_{i})=y^{\left(m\right)}(\tau_{i})$$

where m denotes the order of the derivative. All cases are classified as Birkhoff interpolation except for those with m=0,1,…,K.

#### 3.1.2. Birkhoff Pseudospectral Method Discretization

In practical application scenarios, dynamic models generally do not involve integral operations of order three and above; thus, this paper only elaborates on the first-order and second-order Birkhoff integral matrices.

The Chebyshev–Gauss–Lobatto (CGL) collocation points are adopted, which are defined as the zeros of the derivative of the N-th order Chebyshev polynomial and include the interval endpoints:

(16) $$\tau_{i}=\cos\left(\frac{i\pi }{N}\right),i=0,1,\ldots ,N$$

The higher density of collocation points near the boundaries improves the approximation accuracy of boundary constraints and is well-suited to handling initial/terminal state constraints.

For the optimization problem defined over the time interval $\left[\begin{array}{cc} t_{0} & t_{f} \end{array}\right]$, it is transformed into the standard interval $\left[\begin{array}{cc} -1 & 1 \end{array}\right]$ via the time normalization mapping:

(17) $$\tau =\frac{2}{t_{f}-t_{0}}t-\frac{t_{f}+t_{0}}{t_{f}-t_{0}},$$

and CGL collocation points ${\left\{\tau_{k}\right\}}_{k=0}^{N}$ are set within the interval to discretize the state variables.

Any function f(τ) defined over $\tau \in \left[\begin{array}{cc} -1 & 1 \end{array}\right]$ can be fitted by a series of orthogonal Lagrange polynomials, i.e.,

(18) $$f(\tau )=\sum_{i=0}^{N}f(\tau_{i})\delta (\tau ,i)$$

where δ(τ,i) denotes the Kronecker delta function, which satisfies $\delta (\tau_{j},i)$ = $\delta_{ij}$. Differentiating both sides of the above equation yields

(19) $$\dot{f}(\tau )=\sum_{i=0}^{N}f(\tau_{i})\dot{\delta }(\tau ,i)$$

Let $f=\left[\begin{array}{cccc} f_{0} & f_{1} & \ldots & f_{N} \end{array}\right]$, $D=\left[\dot{\delta }(\tau_{j},i)\right]$,0≤i,j≤N, then Equation (19) can be rewritten as

(20) $$\dot{f}(\tau )=\sum_{i=0}^{N}f(\tau_{i})\dot{\delta }(\tau ,i)=Df$$

The differentiation matrix $D\in R^{\left(N+1)\times (N+1\right)}$ is a core tool for derivative approximation in the pseudospectral method, and it is constructed based on the derivative properties of Chebyshev polynomials.

Birkhoff polynomials are an extension of Lagrange polynomials. The first-order and second-order Birkhoff polynomials of f(τ) are defined as

(21) $$f^{1N}(\tau )=f(\tau_{0})B_{0}^{1}(\tau )+\sum_{k=1}^{N}\dot{f}(\tau_{k})B_{k}^{1}(\tau )$$

(22) $$f^{2N}(\tau )=f(\tau_{0})B_{0}^{2}(\tau )+\sum_{k=1}^{N-1}\dot{f}(\tau_{k})B_{k}^{2}(\tau )+f(\tau_{N})B_{N}^{2}(\tau )$$

where the first-order Birkhoff interpolation basis polynomials $B_{k}^{1}(\tau_{j}),k=1,2,\ldots ,N$ satisfy

(23) $$\begin{array}{c} B_{0}^{1}(\tau_{0})=1,B_{k}^{1}(\tau_{0})=0,k=1,2,\ldots ,N\\ {\dot{B}}_{0}^{1}(\tau_{j})=1,{\dot{B}}_{k}^{1}(\tau_{j})=\delta_{kj},k=1,2,\ldots ,N \end{array}$$

and the second-order Birkhoff interpolation basis polynomials $B_{k}^{2}(\tau_{j}),k=1,2,\ldots ,N$ satisfy

(24) $$\begin{array}{c} B_{0}^{2}(\tau_{0})=1,B_{k}^{2}(\tau_{0})=0,B_{N}^{2}(\tau_{0})=1,k=1,2,\ldots ,N-1\\ B_{0}^{2}(\tau_{N})=0,B_{k}^{2}(\tau_{N})=0,B_{N}^{2}(\tau_{N})=1,k=1,2,\ldots ,N-1\\ {\ddot{B}}_{0}^{2}(\tau_{j})=0,{\ddot{B}}_{k}^{2}(\tau_{j})=\delta_{kj},{\ddot{B}}_{N}^{2}(\tau_{N})=0,k=1,2,\ldots ,N-1 \end{array}$$

Let $B_{k}^{1}(\tau_{i})=B_{ik}^{1}$ and $B_{k}^{2}(\tau_{i})=B_{ik}^{2}$ and then the first-order and second-order Birkhoff integral matrices can be obtained:

(25) $$B_{1}={\left[B_{ij}^{1}\right]}_{0\leq i,j\leq N},B_{1,in}={\left[B_{ij}^{1}\right]}_{1\leq i,j\leq N}$$

(26) $$B_{2}={\left[B_{ij}^{2}\right]}_{0\leq i,j\leq N},B_{2,in}={\left[B_{ij}^{2}\right]}_{1\leq i,j\leq N-1}$$

Combining Equations (20) and (21), the first row of $B_{1}$ is reconstructed as the unit impulse vector $e_{1}=[\begin{array}{ccc} 1 & 0 & \begin{array}{cc} \ldots & 0 \end{array} \end{array}]$ to enforce the satisfaction of the initial boundary constraints. For the first-order differential equation $\dot{f}(\tau )=g(f(\tau ),\tau )$, the input $\dot{F}=\left[\begin{array}{cccc} f(\tau_{0}) & \dot{f}(\tau_{1}) & \ldots & \dot{f}(\tau_{N}) \end{array}\right]$ can be directly converted into the constraint for the state variable $F=\left[\begin{array}{cccc} f(\tau_{0}) & f(\tau_{1}) & \ldots & f(\tau_{N}) \end{array}\right]$ via $B_{1}$:

(27) $$F=B_{1}\dot{F}$$

Similarly, combining Equation (22), the first row and last row of $B_{2}$ are respectively reconstructed as the unit impulse vectors $e_{1}$ and $e_{N}=[\begin{array}{ccc} 0 & \ldots & \begin{array}{cc} 0 & 1 \end{array} \end{array}]$ to enforce the satisfaction of the initial and terminal boundary constraints. For the second-order differential equation $\ddot{f}(\tau )=h(\dot{f}(\tau ),f(\tau ),\tau )$ that satisfies $\dot{f}(\tau_{0})=0$, the input $\dot{F}=\left[\begin{array}{cccc} f(\tau_{0}) & \ddot{f}(\tau_{1}) & \begin{array}{cc} \ldots & \ddot{f}(\tau_{N-1}) \end{array} & f(\tau_{N}) \end{array}\right]$ can be directly converted into the constraint for the state variable F:

(28) $$F=B_{2}\ddot{F}$$

### 3.2. Quaternion and Specific-Force Reconstruction

To meet the discretized input requirement of the Birkhoff pseudospectral method, the quaternions, angular velocity, and specific force need to be projected onto Chebyshev points. Based on the ZUPT detection results, the pedestrian walking trajectory is divided into continuous optimization windows by gait cycles, where each window corresponds to a single gait cycle.

For each window, its time interval is denoted as $\left[\begin{array}{cc} t_{0} & t_{f} \end{array}\right]$, which is mapped to the standard interval $\left[\begin{array}{cc} -1 & 1 \end{array}\right]$ via a linear transformation in Equation (17). At the equal-time-interval moments $t_{k}\;(k=0,1,\ldots ,N)$ within the current window, the specific forces $f_{t_{k}}$ are resampled on the CGL collocation points ${\left\{\tau_{k}\right\}}_{k=0}^{N}$ via the cubic spline interpolation technique, resulting in the aligned ${\tilde{f}}_{\tau_{k}}$.

Since the quaternion $q\in S^{3}$, directly interpolating the quaternion in the Euclidean space will destroy its unitarity, leading to an uneven distribution of angular velocity after interpolation [30,31]. Hence, the Squad interpolation method on the SO(3) group is used for the quaternion reconstruction on the CGL collocation points. For the quaternions $\left\{\begin{array}{cccc} q_{0} & q_{1} & \ldots & q_{N} \end{array}\right\}\subset SO(3)$, an auxiliary point $s_{i}$ is assigned to each interpolation point to smooth the angular velocity of the interpolation curve. $s_{i}$ is defined by the logarithmic mapping difference of adjacent quaternions:

(29) $$s_{i}=q_{i}⊗\exp(-\frac{\log(q_{i}^{-1}⊗q_{i+1})+\log(q_{i-1}^{-1}⊗q_{i})}{4})$$

When computing auxiliary points for $q_{i}$, the adjacent quaternions $q_{-1}$ and $q_{N+1}$ are undefined at the endpoints i=0 and i=N. These virtual quaternions are constructed via spherical mirroring on $S^{3}$: $q_{-1}$ is obtained by reflecting $q_{1}$ across the boundary quaternion $q_{0}$, while $q_{N+1}$ is formed by reflecting $q_{N-1}$ across $q_{N}$, preserving the spherical geometric properties of unit quaternions.

The Slerp interpolation is given by

(30) $$S(q_{a},q_{b},\beta )=\frac{\sin((1-\beta )\theta )}{\sin\theta }q_{a}+\frac{\sin(\beta \theta )}{\sin\theta }q_{b}$$

where $\theta =\arccos(\left|q_{a}\cdot q_{b}\right|)$ and β is defined as a normalized interpolation parameter confined to the interval $\left[\begin{array}{cc} 0 & 1 \end{array}\right]$. Then, the Squad interpolation within the interval $t\in \left[\begin{array}{cc} t_{i} & t_{i+1} \end{array}\right]$ is given by

(31) $$q(t)=S(S(q_{i},q_{i+1},\alpha ),S(s_{i},s_{i+1},\alpha ),2\alpha (1-\alpha ))$$

where $\alpha =\frac{t-t_{i}}{t_{i+1}-t_{i}}$.

The quaternion obtained through the above transformation is denoted as $\tilde{q}$, and ${\tilde{C}}_{b}^{n}$ is the rotation matrix corresponding to $\tilde{q}$.

### 3.3. Velocity–Position Optimization

Velocity–position optimization is a key process that estimates the position $p^{n}$, velocity $v^{n}$, and accelerometer bias $b_{a}$ of the carrier within the optimization time interval $\left[\begin{array}{cc} t_{0} & t_{f} \end{array}\right]$, based on the discrete specific force ${\tilde{f}}_{\tau_{k}}$ and rotation matrix ${\tilde{C}}_{b}^{n}$. Given the known attitude sequence, this process converts the complex nonlinear coupling problem into a relatively simple optimization problem and achieves high-precision trajectory reconstruction via the global discretization framework of the Birkhoff pseudospectral method.

In the navigation frame, the continuous-time dynamic equation of the carrier’s motion can be expressed as shown in Equations (2) and (3). In Equation (2), the true specific force f can be approximated by its corresponding state estimate $\hat{f}=\tilde{f}-b_{a}$. The velocity–position optimization problem is defined as a constrained dynamic optimization problem, where the system state variable X is defined as $\left[\begin{array}{cc} v^{n} & \begin{array}{cc} p^{n} & b_{a} \end{array} \end{array}\right]$, and the control variable U is defined as $\left[\begin{array}{cc} \tilde{f} & {\tilde{C}}_{b}^{n} \end{array}\right]$.

The objective function of velocity–position optimization aims to balance boundary prior information, dynamic constraints, and smoothing terms. Its mathematical formulation is as follows:

(32) $$\underset{p(t),v(t),b_{a}}{\min}\;J_{1}=J_{x0}+J_{z}+J_{s}$$

The prior term $J_{x0}$ in Equation (32) is designed to deal with the boundary conditions provided by ZUPT, as follows:

(33) $$J_{x0}=\lambda_{x0}({\left\|p(\tau_{0})-p_{0}\right\|}^{2}+{\left\|v(\tau_{f})-v_{f}\right\|}^{2}+{\left\|v(\tau_{0})-v_{0}\right\|}^{2})$$

where $p_{0}$, $v_{0}$ and $v_{f}$ represent the initial position, initial velocity, and terminal velocity transferred from the previous optimization window, respectively. In practical implementations, the initial velocity $v_{0}$ and terminal velocity $v_{f}$ are typically set to 0. Through Equation (33), we can enforce the initial state of the optimized trajectory to be consistent with physical rules. Here, the designed parameter $\lambda_{x0}$ is the prior term’s weight coefficient: selected for trajectory parameterization, it balances ZUPT boundary constraint credibility and trajectory optimization flexibility—ensuring constraint reliability and the initial state’s consistency with physical rules.

The measurement term $J_{z}$ is designed to minimize the difference between the raw specific force measured by the IMU and the theoretical specific force derived from the numerically differentiated trajectory, as follows:

(34) $$J_{z}=\lambda_{z}\sum_{i=0}^{k}{\left({\dot{v}}_{k}^{n}-{\tilde{C}}_{b,k}^{n}({\tilde{f}}_{\tau_{k}}-b_{a,k})-g^{n}\right)}^{T}R_{a}^{-1}\left({\dot{v}}_{k}^{n}-{\tilde{C}}_{b,k}^{n}({\tilde{f}}_{\tau_{k}}-b_{a,k})-g^{n}\right)$$

where $R_{a}$ is the accelerometer noise covariance matrix, and $\lambda_{z}$ denotes the weighting coefficient of the measurement term. It is adjusted to match the noise characteristics of MEMS-IMUs, thereby effectively reducing deviations between the measured and theoretical acceleration data.

Since the accelerometer bias is treated as a state variable, its variation is physically slow and does not fluctuate sharply in a short time. Therefore, a smoothing term $J_{s}$ is added to constrain the estimated bias to maintain continuous and smooth variation in the time dimension:

(35) $$J_{s}=\lambda_{s}\sum_{i=0}^{k-1}{\left\|△b_{a,k}\right\|}^{2}$$

here, $\Delta b_{a}$ denotes the variation of the accelerometer bias, and $\lambda_{s}$ is the weight coefficient of the smoothing term. $\lambda_{s}$ is tuned to constrain the amplitude of $\Delta b_{a}$, thereby restricting abrupt fluctuations of the accelerometer bias—this ensures the temporal continuity of the bias and its consistency with the slow time-varying physical characteristic.

The periodicity of pedestrian gait provides natural boundary conditions for zero-velocity moments. By using the ZUPT algorithm to accurately detect the static moment at the end of the stance phase, the long-endurance navigation trajectory can be divided into a series of continuous optimization windows. For each window, its boundary conditions are given by Equation (33). Based on the above discretization and window division, the velocity–position trajectory optimization problem can be transformed into a finite-dimensional NLP problem of the following form:

(36) $$\begin{array}{c} \underset{p(\tau ),v(\tau ),b_{a}}{\min}\;J_{1}=J_{x0}+J_{z}+J_{s}\\ s.t.\;{\dot{v}}^{n}(\tau_{k})={\tilde{C}}_{b,k}^{n}({\tilde{f}}_{\tau_{k}}-b_{a,k})+g^{n},k=0,\ldots ,N\;\\ {\dot{p}}^{n}(\tau_{k})=v^{n}(\tau_{k})\\ p(\tau_{0})=p_{0}\\ v(\tau_{0})=0,v(\tau_{f})=0 \end{array}$$

Based on the properties of the Birkhoff integral matrix and pedestrian boundary conditions, $v^{n}$ and $p^{n}$ can be approximated as

(37) $$v_{j}^{n}\approx \sum_{j=0}^{N-1}B_{1,kj}{\dot{v}}^{n}(\tau_{k})$$

(38) $$p_{j}^{n}\approx \sum_{j=0}^{N-1}B_{1,kj}{\dot{p}}^{n}(\tau_{k})$$

Then the dynamic constraint in Equation (36) can be rewritten as

(39) $$v_{j}^{n}-\gamma \sum_{k=0}^{N}B_{1,kj}\left[{\tilde{C}}_{b,k}^{n}({\tilde{f}}_{\tau_{k}}-b_{a,k})-g^{n}\right]=0$$

(40) $$p_{j}^{n}-\gamma \sum_{j=0}^{N-1}B_{1,kj}v_{j}^{n}=0$$

here, γ is defined as the time scaling coefficient, with its value given by $\frac{t_{f}-t_{0}}{2}$.

To improve the robustness of the optimization problem and avoid infeasible solutions caused by measurement noise or model errors, slack variables are introduced to convert the above dynamic hard constraints into soft constraints. The constraint conditions after slack processing are as follows:

(41) $${\sum_{j=0}^{N}\left\|v_{j}^{n}-\gamma \sum_{k=0}^{N}B_{1,kj}\left[{\tilde{C}}_{b,k}^{n}({\tilde{f}}_{\tau_{k}}-b_{a,k})-g^{n}\right]\right\|}^{2}\leq \varepsilon_{v}$$

(42) $${\sum_{j=0}^{N}\left\|p_{j}^{n}-\gamma \sum_{j=0}^{N-1}B_{1,kj}v_{j}^{n}\right\|}^{2}\leq \varepsilon_{p}$$

here, $\varepsilon_{v}$ and $\varepsilon_{p}$ are the velocity slack coefficient and position slack coefficient, respectively.

For the constraints after slack processing, a penalty term $J_{p}$ needs to be added to the objective function to penalize deviations from the dynamic constraints. The expression of this penalty term is as follows:

(43) $$J_{p}=\lambda_{p}\sum_{k=0}^{N-1}\left({\left\|{\dot{p}}_{k}^{n}-v_{k}^{n}\right\|}^{2}+{\left\|{\dot{v}}_{k}^{n}-{\tilde{C}}_{b,k}^{n}({\tilde{f}}_{\tau_{k}}-b_{a,k})-g^{n}\right\|}^{2}\right)$$

where $\lambda_{p}$ is the weight of the penalty term. It is tuned via experimental tuning to effectively penalize deviations from dynamic constraints, while ensuring the robustness of the optimization problem and preventing infeasible solutions induced by MEMS-IMU measurement noise or model mismatches.

The values of these weighting coefficients are set via systematic experimental tuning, ensuring that the gradients of all terms in the objective function remain within comparable orders of magnitude, thereby promoting stable and balanced convergence during optimization.

Thus, the discretized velocity–position trajectory optimization problem can be formulated as the following NLP problem:

(44) $$\begin{array}{c} \underset{p^{n},v^{n},b_{a}}{\min}\;J_{1}=J_{x0}+J_{z}+J_{s}+J_{p}\\ s.t.\;{\sum_{j=0}^{N}\left\|v_{j}^{n}-\gamma \sum_{k=0}^{N}B_{1,kj}\left[{\tilde{C}}_{b,k}^{n}({\tilde{f}}_{\tau_{k}}-b_{a,k})-g^{n}\right]\right\|}^{2}\leq \varepsilon_{v}\;\\ {\sum_{j=0}^{N}\left\|p_{j}^{n}-\gamma \sum_{j=0}^{N-1}B_{1,kj}v_{j}^{n}\right\|}^{2}\leq \varepsilon_{p}\\ p(\tau_{0})=p_{0}\\ v(\tau_{0})=0,v(\tau_{f})=0 \end{array}$$

Algorithm 1 outlines the core procedures of the pedestrian navigation trajectory optimization method presented in this work. The method operates in an iterative workflow, as detailed below:
**Algorithm 1:** Birkhoff pseudospectral optimization algorithm initialized via ZUPT/EKF**Input:** $\omega_{ib}^{b},f_{ib}^{b}$. **Output:** Globally optimized state sequence X.
Initialize states as $p_{0}^{n}=0$, $v_{0}^{n}=0$, $q_{b}^{n}(0)=q_{0}$.
Compute the Birkhoff integral matrix $B_{1}$.
Compute the zero-velocity intervals $W=\left[\begin{array}{cccc} W_{0} & W_{1} & \ldots & W_{k} \end{array}\right]$. for i=0,…,k
Estimate $x_{i}=[\begin{array}{ccc} {\hat{p}}^{n} & {\hat{v}}^{n} & q_{b}^{n} \end{array}]$ via EKF.
Compute $U_{i}=[\begin{array}{cc} \tilde{f} & {\tilde{C}}_{b}^{n} \end{array}]$ and $X_{i}^{\left\{0\right\}}=[\begin{array}{ccc} p^{n} & v^{n} & b_{a} \end{array}]$ via Equation (31) and cubic spline interpolation.
for j=1,…,MaxIter
Compute $X_{i}^{\left\{j\right\}}$ via Equation (44) by employing the IPOPT solver.
Compute the primal residual $r_{p}$ and the dual residual $r_{d}$.
$\mathrm{if}\;r_{p}\leq \varepsilon_{rp}\;\mathrm{and}\;r_{d}\leq \varepsilon_{rd}$
$X_{i}=X_{i}^{\left\{j\right\}}$.
break
end for end for


## 4. Simulation Experiments

### 4.1. Simulation Settings

This section constructs a simulation scenario in which a pedestrian equipped with a single-foot-mounted IMU walks along a closed horizontal square trajectory. The scenario is designed to conduct a comparative performance analysis between the traditional ZUPT-aided EKF algorithm and the proposed ZUPT-aided Birkhoff pseudospectral optimization algorithm.

In the simulation, the pedestrian is assumed to walk along straight-line segments (forming the sides of the square trajectory) on a horizontal plane. The initial position and velocity of the IMU-mounted foot are set as

(45) $$p^{n}=\left[\begin{array}{ccc} 0 & 0 & 0 \end{array}\right],\;v^{n}=\left[\begin{array}{ccc} 0 & 0 & 0 \end{array}\right],$$

the Euler angles are set as

(46) $$\theta_{n,L}^{b}=\left[\begin{array}{ccc} \varphi_{nb} & \psi_{nb} & \theta_{nb} \end{array}\right],$$

where $\varphi_{nb}$, $\psi_{nb}$ and $\theta_{nb}$ represent the roll, yaw, and pitch angles, respectively.

In pedestrian navigation, a single gait cycle is divided into the swing phase and the stance phase. This characteristic forms the basis for the application of techniques such as ZUPT. During the stance phase, the foot is typically assumed to be instantaneously stationary relative to the ground, which constitutes the physical foundation for zero-velocity updates. In the k-th swing phase, the positional displacement of the foot relative to its location at the end of the previous stance phase can be modeled as $\Delta p=\left[\begin{array}{ccc} p_{n} & p_{e} & p_{u} \end{array}\right]$.

(47) $$p_{n}=l_{s}\left\{1-\cos\left[\pi (t-kT)/t_{u}\right]\cos(\psi_{0})\right\}$$

(48) $$p_{e}=l_{s}\left\{1-\cos\left[\pi (t-kT)/t_{u}\right])\sin(\psi_{0})\right\}$$

(49) $$p_{u}=l_{h}\left[1-\cos(2\pi (t-kT)/t_{u})\right]$$

where $l_{s}$ represents the single-step stride length, $t_{u}$ and T denote the duration of the swing phase and the complete gait cycle, respectively, and $l_{h}$ indicates the maximum clearance height of the foot movement. After constructing the position trajectory function of the foot during the swing phase based on these parameters, the theoretical velocity and acceleration in the n-frame can be obtained by differentiating with respect to time.

Similarly, the foot’s attitude $\theta_{n,L}^{b}$ during each stance phase can be set as

(50) $$\begin{array}{c} \varphi_{nb}=0\;\psi_{nb}=\psi_{0}\\ \theta_{nb}=\theta_{\max}\left\{1-\cos\left[2\pi \left(t-kT\right)/t_{u}\right]\right\} \end{array}$$

where $\theta_{\max}$ denotes the maximum elevation angle during walking. In the stance phase, the position, velocity, and attitude are consistent with the state at the end of the final swing phase. The angular velocity formula can be derived via calculation:

(51) $$\omega =\left[\begin{array}{c} 0\\ 0\\ {\dot{\theta }}_{nb} \end{array}\right]=\left[\begin{array}{c} 0\\ 0\\ \frac{\theta_{\max}}{t_{u}}\sin\left[\frac{2\pi }{t_{u}}(t-kT)\right] \end{array}\right]$$

**Figure 2:**
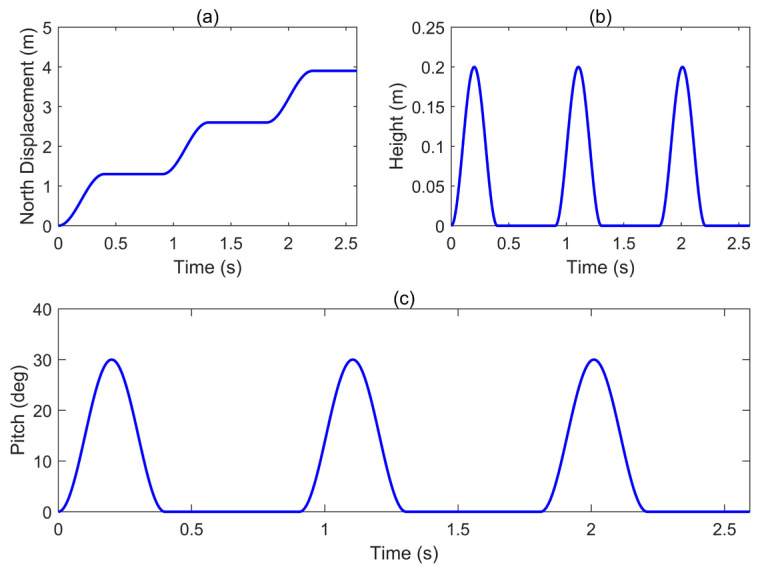
Simulated pedestrian walking states over three gait cycles: (**a**) northward displacement, (**b**) upward displacement, (**c**) pitch angle.

**Figure 3:**
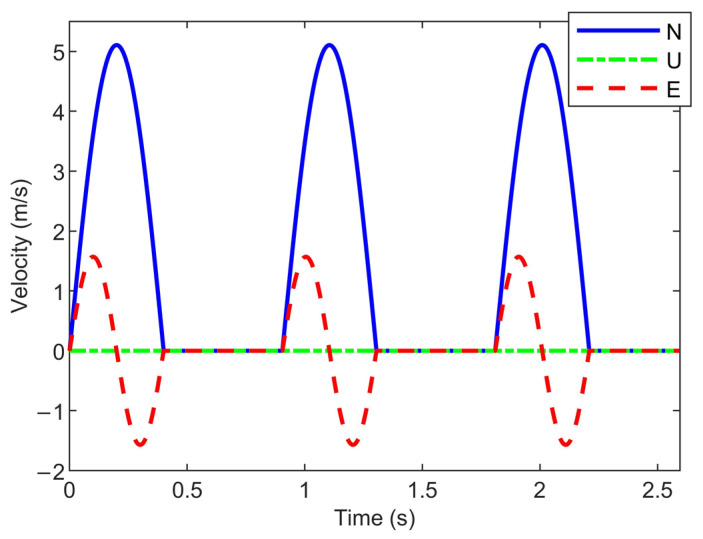
Simulated three-axis velocity during south-to-north movement over three gait cycles.

Figure 2 simulates the trajectory of the pedestrian moving from south to north in the simulation, while Figure 3 presents the three-axis velocity of the pedestrian during the south-to-north movement:

To enable the pedestrian to walk along a square trajectory, a turning maneuver is implemented, which alters only the heading angle without changing the position. If the initial heading at the start of the turn is $\psi_{0}$, the attitude of the left foot is given by

(52) $$\begin{array}{c} \varphi_{nb}=0\;\theta_{nb}=0\\ \psi_{nb}=\frac{\pi }{4}\left\{1-\cos\left[\pi \left(t-t_{0}\right)/t_{d}\right]\right\}+\psi_{0} \end{array}$$

where $t_{d}$ is the turning duration, and $t_{0}$ denotes the start time of the turn. During the turning process, the position remains unchanged, and the velocity is set to zero. The angular velocity during the turn can be calculated as

(53) $$\omega =\left[\begin{array}{c} 0\\ {\dot{\psi }}_{nb}\\ 0 \end{array}\right]=\left[\begin{array}{c} 0\\ \frac{\pi }{4t_{d}}\sin\left[\frac{\pi }{t_{d}}(t-t_{0})\right]\\ 0 \end{array}\right]$$

### 4.2. Experimental Results

**Figure 4:**
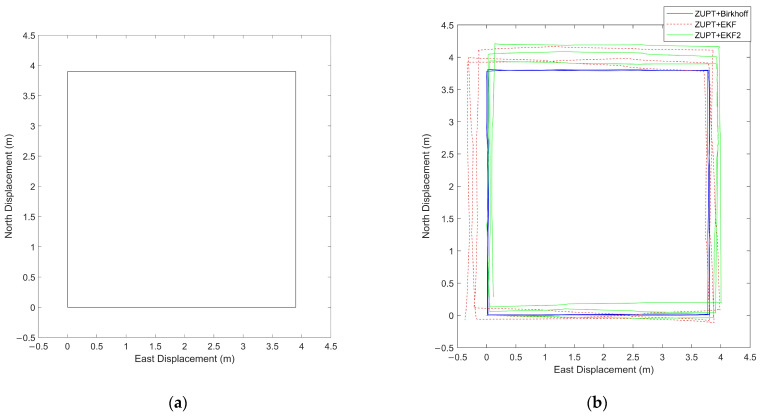
(**a**) Simulated horizontal trajectory; (**b**) planar trajectories generated by the Birkhoff pseudospectral method, the Extended Kalman Filter (EKF) algorithm, and the Second−Order Extended Kalman Filter (EKF2) algorithm.

In the simulation experiment, the pedestrian is set to walk along a square trajectory with a side length of 3$l_{s}$, completing three laps before returning to the starting point and coming to a stop. The simulation parameters are listed in Table 1. The IMU sampling frequency is set to 200 Hz. Assuming an initial heading angle of zero, the pedestrian makes a 90° left turn at each vertex of the square. The total travel distance is 46.8 m, and the total duration is 45.1 s. The reference trajectory within the horizontal plane is shown in Figure 4a.

This study compares the proposed Birkhoff pseudospectral method with two established filtering baselines: the EKF and the Second-Order Extended Kalman Filter (EKF2). The EKF2 serves as a more rigorous benchmark for pedestrian navigation with ZUPT. Unlike the standard EKF, the EKF2 operates on the error state, not the full navigation state. This framework avoids singularities in attitude representation, reduces linearization errors, and improves numerical stability [32]. This stability is especially critical in foot-mounted IMU applications, where frequent stance-phase ZUPT corrections are applied.

For fair comparison, all algorithms adopt a unified attitude input, specifically the attitude estimated by the EKF. The EKF2 only observes velocity states, with Rauch–Tung-Striebel (RTS) smoothing integrated. As a classic post-processing global optimization method for recursive filters, RTS smoothing is employed herein to optimize the EKF2-estimated trajectory by utilizing full-time-domain measurement information [33]. This enables a valid performance comparison between the EKF2 and the Birkhoff pseudospectral method in the global optimization framework.

To evaluate the trajectory estimation accuracy of the Birkhoff pseudospectral method under noise interference, white noise with different power spectral densities was added to the accelerometer of the IMU in the simulation. Under given attitude error conditions, the performance of the Birkhoff pseudospectral method with different numbers of collocation points, the EKF algorithm, and the EKF2 algorithm in position estimation was compared, focusing on the final position error and the root mean square error (RMSE) over the entire trajectory. Table 2 summarizes the comparison results of the position RMSE and final position error for the three algorithms under different numbers of collocation points and different noise levels.

Under identical attitude error conditions, the RMSE and final position error of the EKF algorithm are approximately 10 times greater than those of the Birkhoff pseudospectral method. Meanwhile, the EKF2 still yields RMSE and final position error values that are roughly 3–4 times higher than the corresponding results of the Birkhoff method. Even in scenarios with an accelerometer noise intensity as high as ${0.1\;m/s}^{2}/\sqrt{\mathrm{Hz}}$, the Birkhoff pseudospectral method can still maintain reliable positioning accuracy.

This indicates that the state estimation accuracy of the Birkhoff pseudospectral method under noisy measurement conditions is significantly superior to that of the EKF algorithm and EKF2 algorithm. This advantage stems from the global optimization nature of the pseudospectral method: by discretizing the entire time domain with collocation points and employing orthogonal polynomial approximations, it transforms the dynamic differential constraints into algebraic constraints. Consequently, when solving the NLP problem, it can effectively smooth out error fluctuations induced by noise. This principle inherently avoids the problem of error accumulation over time, which is common in recursive filters like the EKF.

This advantage of the Birkhoff pseudospectral method stems from its inherent full-time-domain global optimization property. The algorithm discretizes the entire time domain via collocation points and converts dynamic differential constraints into algebraic constraints with orthogonal polynomial approximation. It can thus effectively smooth out noise-induced error fluctuations when solving NLP problems. This approach fundamentally avoids the time-dependent error accumulation issue of the EKF2-RTS combined algorithm. The EKF2-RTS still relies on step-by-step recursive estimation for state calculation in the filtering stage, and RTS smoothing only performs post-processing correction on the recursively obtained state sequence, rather than reconstructing the trajectory from the source.

To investigate the impact of the number of collocation points on algorithm performance, a comparative experiment was conducted with different collocation-point settings. Theoretically, increasing the number of collocation points helps improve discretization accuracy; however, in practical, noisy scenarios, an excessive number of points may lead to overfitting. The experimental results show that even with a relatively low number of collocation points, the Birkhoff pseudospectral method still achieves significantly higher estimation accuracy than the EKF and the EKF2. Furthermore, the RMSE decreases with the increase of N, indicating that insufficient collocation points introduce truncation errors in approximating highly dynamic motions. Therefore, a suitable number of collocation points should be chosen by balancing positioning accuracy and computational cost in practical applications. Nevertheless, the proposed method maintains stable and superior estimation performance over a wide range of collocation points. It exhibits strong numerical robustness and engineering practicality. Figure 4b presents a comparison of the planar trajectories generated by the Birkhoff pseudospectral method with N=60 collocation points, the EKF algorithm, and the EKF2 algorithm, under an accelerometer noise intensity of ${0.03\;m/s}^{2}/\sqrt{\mathrm{Hz}}$.

## 5. Real-World Experiments

To comprehensively evaluate the real-world performance of the proposed algorithm, this section conducts experiments in both a controlled indoor environment and an open outdoor scenario. The proposed method is compared with the EKF algorithm and the EKF2 algorithm. All comparisons are based on the same sensor data, attitude information, and ZUPT detection results to ensure fairness, with a focus on evaluating the algorithms’ performance in terms of position estimation accuracy and accumulated travel error.

In the experiments, a commercial IMU (MTi-G-710 from Xsens, Enschede, Netherlands) was securely attached to the tester’s foot. Based on the experience gained from the preliminary simulations, the number of collocation points for the Birkhoff pseudospectral method was uniformly set to 60 to prevent inadequate approximation of the motion states due to an insufficient number of points.

**Figure 5:**
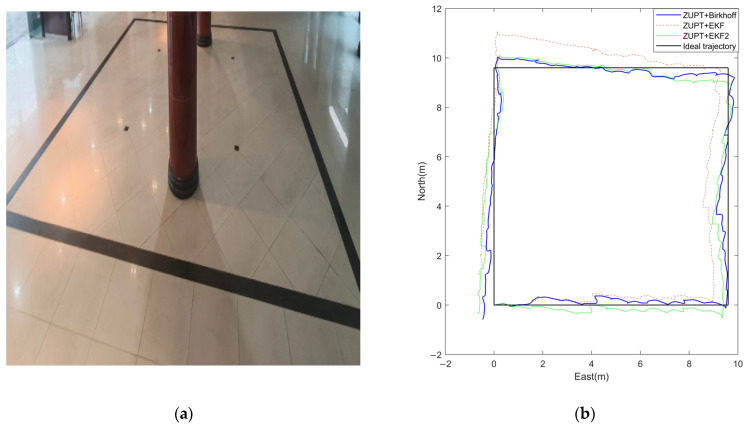
(**a**) Indoor walking trajectory; (**b**) comparison of estimated trajectories from the Birkhoff pseudospectral method, the EKF algorithm, and the EKF2 algorithm in the indoor scenario.

Indoor Experiment: The tester walked along a rectangular closed path with a side length of 9.6 m. The actual scene is shown in Figure 5a. This environment, effectively shielded from satellite signals, was used to validate the algorithm’s pure inertial navigation performance.

**Figure 6:**
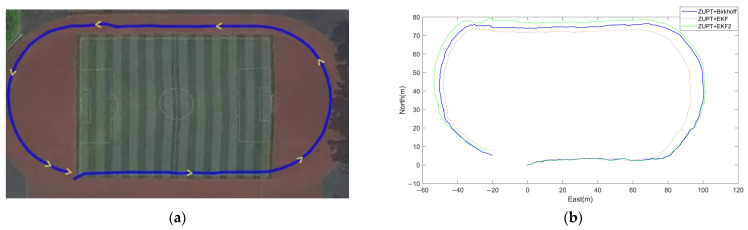
(**a**) Outdoor walking trajectory under GPS reference; (**b**) Comparison of estimated trajectories from the Birkhoff pseudospectral method, the EKF algorithm, and the EKF2 algorithm in the outdoor scenario.

Outdoor Experiment: The tester walked one complete lap along the innermost lane of a standard 400-m-perimeter athletics track. GPS data was collected simultaneously to serve as the reference trajectory for evaluating positioning accuracy. The actual movement trajectory under GPS reference is shown in Figure 6a.

**Figure 7:**
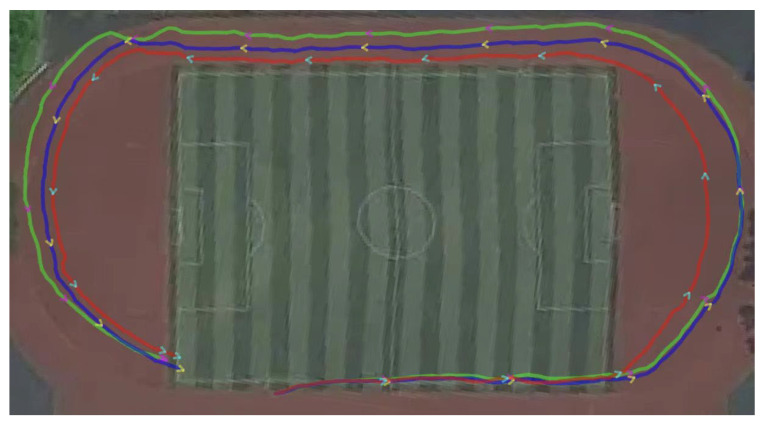
Georeferenced visualization of estimated trajectories (solid blue line: Birkhoff pseudospectral method; solid green line: EKF2; solid red line: EKF).

Figure 5b and Figure 6b present the estimated planar motion trajectories generated by the three algorithms in the indoor and outdoor environments, respectively. Visually, the trajectory produced by the proposed algorithm aligns more closely with the reference path, exhibiting significantly smoother motion and less divergence compared to the EKF results and the EKF2. Notably, Figure 7 overlays the outdoor estimated trajectory onto a satellite map, providing further evidence of the proposed algorithm’s advantage in mitigating cumulative drift and enhancing the global consistency of the estimated path.

For precise quantitative comparison, Table 3 summarizes multiple error metrics from the indoor and outdoor experiments, including the start-point error, final position error, absolute distance error, and relative distance error.

In both indoor and outdoor experiments, the proposed algorithm outperforms both the EKF and EKF2.

It significantly reduces the final position error and relative distance error compared with the EKF, with reduction rates of approximately 70% and 90% in indoor and outdoor scenarios, respectively.

Compared with the EKF2, the proposed method achieves a comparable final position error in indoor tests and reduces the relative distance error by about 20% compared with the EKF2. In the outdoor scenario, it decreases the relative distance error by roughly 70% and the final position error by approximately 15%.

These experimental results demonstrate that the proposed Birkhoff pseudospectral optimization algorithm effectively utilizes global gait cycle information and suppresses the cumulative noise errors of MEMS-IMU. It outperforms both the traditional ZUPT-aided EKF and the advanced EKF2 in trajectory smoothness, distance estimation accuracy, and final position precision.

## 6. Conclusions

This paper proposes a trajectory optimization method for pedestrian inertial navigation based on the Birkhoff pseudospectral method. Drawing on initial navigation states from ZUPT and EKF, the method discretizes the state trajectory via Chebyshev collocation points and solves a global optimization problem using the Birkhoff pseudospectral method.

To validate the effectiveness of the proposed algorithm and its applicability to pedestrian navigation, both simulation and physical experiments are designed and conducted. The experimental results demonstrate that the proposed algorithm outperforms both the traditional ZUPT-aided EKF and the advanced EKF2, achieving significant reductions in final position error and relative distance error. Specifically, the relative distance error is reduced by approximately 70% and 90% compared with the EKF in indoor and outdoor scenarios, respectively, and clear accuracy improvements are also obtained over the EKF2.

Theoretically, the results originate from the time-domain global optimization property of the Birkhoff pseudospectral method. Different from the recursive estimation of EKF/EKF2 that tends to induce error accumulation, the proposed method performs centralized optimization on short-duration pedestrian trajectories. It corrects local deviations caused by IMU noise from a global perspective and effectively suppresses noise interference in navigation within a short time.

Future work will explore integrating this method with other advanced estimation frameworks, such as factor graph optimization, to further enhance the performance of pedestrian navigation systems in complex environments.

## Figures and Tables

**Table 1:** Simulation parameter configuration.

Parameter	Notation	Value
Stride length	$l_{s}$	1.3 m
Max height	$l_{h}$	0.2 m
Swing time	$t_{u}$	0.4 s
Max pitch	$\theta_{\max}$	30°
Turning time	$t_{d}$	0.3 s

**Table 2:** Position estimation errors under different accelerometer noise levels: comparison between the Birkhoff pseudospectral method, the EKF, and the EKF2.

Method	Accelerometer Noise Intensity $({m/s}^{2}/\sqrt{\mathrm{Hz}}$)	Root Mean Square Error (RMSE) (m)	Position Error (m)
Birkhoff N = 20	0.01	0.1896	0.0116
	0.02	0.2211	0.0244
	0.03	0.2088	0.0254
	0.1	0.3144	0.2430
Birkhoff N = 40	0.01	0.1547	0.0107
	0.02	0.1504	0.0229
	0.03	0.1378	0.0274
	0.1	0.2614	0.2323
Birkhoff N = 60	0.01	0.1198	0.0126
	0.02	0.1146	0.0273
	0.03	0.1035	0.0332
	0.1	0.2572	0.3012
EKF	0.01	0.7787	0.3546
	0.02	0.8645	0.2720
	0.03	0.9032	0.4581
	0.1	1.1674	0.9594
EKF2	0.01	0.3751	0.1042
	0.02	0.4287	0.2157
	0.03	0.5536	0.2953
	0.1	0.8243	0.5276

**Table 3:** Error performance comparison between the Birkhoff pseudospectral method, the EKF algorithm, and the EKF2 algorithm.

Method	Final Position Error (m)	Absolute Distance Error (m)	Relative Distance Error (%)
ZUPT + Birkhoff (indoor)	0.7476	0.2763	0.7196%
ZUPT + EKF2 (indoor)	0.7134	0.3394	0.8839%
ZUPT + EKF (indoor)	1.4316	0.9892	2.5761%
ZUPT + Birkhoff (outdoor)	20.9032	1.7061	0.4265%
ZUPT + EKF2 (outdoor)	22.9734	5.2184	1.3046%
ZUPT + EKF (outdoor)	22.4810	16.4777	4.12%

## Data Availability

The data are unavailable due to privacy restrictions.
